# Bibliographical Notices

**Published:** 1856-08

**Authors:** 


### BIBLIOGRAPHICAL NOTICES.

*The Microscope and its Revelations.* By WILLIAM B. CARPENTER, M. D., F. R. S., F. G. S., &c. *With an appendix containing the Applications of the Microscope to Clinical Medicine.* By FRANCIS GURNEY SMITH, M. D., Professor of the Institutes of Medicine in the Medical Department of Pennsylvania College, &c. *Illustrated by four hundred and thirty-four engravings on wood.* Philadelphia: Blanchard & Lea, 1856.

The use of the microscope has become so general throughout our whole country during the last few years, that we are confident the publication of the above work, containing an account of its various forms and appurtenances, together with a full description of the microscopic conditions of vegetable and animal life, made manifest by its assistance, cannot be otherwise than most favorably received by numbers, both in and out of the profession. The author is one of the most, if not the most attractive of living writers upon scientific subjects. Whatever he has undertaken, he has well performed. Everything he writes becomes at once popular; the merit of all his works being, that he presents his readers in clear and apposite language, and filtered through a sound and practical intellect, everything that is contemporarily known upon the subject he is discussing. In this particular we doubt whether he has his equal in the world. As an original investigator, his claims are less prominent, though even in this respect, he has done much to signalize himself. To the various departments of microscopic enquiry in particular, it is well known he has ardently devoted himself for many years; there are few, in fact, who have contributed more to its advancement. As the result of these studies, the present work, which was announced several years ago, and which we take the present occasion to recommend warmly to our readers, is now before us.

The work is divided into twenty chapters, of which the first five describe the optical principles, construction, accessory apparatus and management of the microscope, together with the proper modes of preparing and mounting objects. The following thirteen chapters are devoted to the description of the microscopic forms of vegetable and animal life, of the protophytes, of the minute structure of higher cryptogamia, of phanerogamic plants, protozoa, &c. The nineteenth is on the application of the microscope to geology, and the twentieth on mineral objects, polarization, &c.

Regarding the application of the microscope, its use by beginners and the amount of space allotted by the author to the various departments of enquiry, the preface contains the following remarks:—

"In treating of the Applications of the Microscope, the Author has constantly endeavored to meet the wants of those who come to the study of the minute forms of Animal and Vegetable life with little or no previous scientific preparation, but who desire to gain something more than a mere *sight* of the objects to which their observation may be directed. Some of these may perhaps object to the general tone of his work as too highly pitched, and may think that he might have rendered his descriptions simpler by employing fewer scientific terms. But he would reply to such, that he has had much opportunity of observing, among the votaries of the Microscope, a desire for such information as he has attempted to convey (of the extent of which desire, the success of the 'Quarterly Journal of Microscopical Science' is a very gratifying evidence); and that the use of scientific terms cannot be dispensed with, since there are no others in which the facts can be expressed. As he has made a point of explaining these, in the places where they are first introduced, he cannot think that any of his readers need find much difficulty in apprehending their meaning.

The proportion of space allotted to the various departments, has been determined, not so much by their Physiological importance, as by their special interest to the Microscopist; and the remembrance of this consideration will serve to account for much that might otherwise appear strange. The Author has thought it particularly needful to restrain himself, in treating of certain very important subjects which are fully discussed in treatises expressly devoted to them (such, for example, as the structure of Insects, and the Primary Tissues of Vertebrata), in order that he might give more space to those on which no such sources of information are accessible. For the same reason he has omitted all reference to the applications of the Microscope to Pathological inquiry; a subject which would interest only one division of his readers, and on which it would have been impossible for him to compress, within a sufficiently narrow compass, a really useful summary of what such readers can readily learn elsewhere. So again, the application of the Microscope to the detection of Adulterations in Food, &c., is a topic of such a purely special character, and must be so entirely based on detailed descriptions of the substances in question, that he has thought it better to leave this also untouched.

It has been the Author's object throughout, to guide the possessor of a Microscope to the *intelligent* study of any department of Natural History, that his individual tastes may lead him to follow out, and his particular circumstances may give him facilities for pursuing. And he has particularly aimed to show, under each head, how small is the amount of reliable knowledge already acquired, compared with that which remains to be attained by the zealous and persevering student. Being satisfied that there is a large quantity of valuable *Microscope-power* at present running to waste in this country,—being applied in such desultory observations as are of no service whatever to science, and of very little to the mind of the observer,—he will consider the production of this Manual as well repaid, if it should tend to direct this power to more systematic labors, in those fertile fields which only await the cultivator to bear abundant fruit."

The Educational Value and Uses of the Microscope are largely dilated upon in the Introduction. Of these, the author very naturally and very correctly, we think, has a very high opinion. The good sense of the following remarks will commend themselves to our readers :—

"It cannot be too strongly or too constantly kept in view, that the value of the results of Microscopic inquiry will depend far more upon the sagacity, perseverance, and accuracy of the observer, than upon the elaborateness of his instrument. The most perfect Microscope ever made, in the hands of one who knows not how to turn it to account, is valueless; in the hands of a careless, a hasty, or a prejudiced observer, it is worse than valueless, as furnishing new contributions to the already large stock of errors that pass under the guise of scientific truths. On the other hand, the least costly Microscope that has ever been constructed, how limited soever its powers, provided that it gives no *false* appearances, shall furnish to him who knows what *may* be done with it, a means of turning to an account, profitable alike to science and to his own immortal spirit, those hours which might otherwise be passed in languid *ennui*, or in frivolous or degrading amusements, and even of immortalizing his name by the discovery of secrets in Nature as yet undreamed of. A very large proportion of the great achievements of Microscopic research that have been noticed in the preceding outline, have been made by the instrumentality of microscopes which would be generally condemned in the present day as utterly unfit for any scientific purpose; and it cannot for a moment be supposed, that the field which Nature presents for the prosecution of inquiries with instruments of comparatively limited capacity, has been in any appreciable degree exhausted. On the contrary, what *has been* done by these and scarcely superior instruments, only shows how much there is *to be* done. The author may be excused for citing, as an apposite example of his meaning, the curious results he has recently obtained from the study of the development of the *Purpura lapillus* (rockwhelk), which will be detailed in their appropriate place (Chap. XII); for these were obtained almost entirely by the aid of *single lenses*, the Compound Microscope having been only occasionally applied to, for the verification of what had been previously worked out, or for the examination of such minute details as the power employed did not suffice to reveal.

But it should be urged upon such as are anxious to do service to science, by the publication of discoveries which they suppose themselves to have made with comparatively imperfect instruments, that they will do well to refrain from bringing these forward, until they shall have obtained the opportunity of verifying them with better. It is, as already remarked, when an object is *least* clearly seen, that there is *most* room for the exercise of the imagination; and there was sound sense in the reply once made by a veteran observer, to one who had been telling him of wonderful discoveries which another was said to have made '*in spite* of the badness of his Microscope,'—'No, sir, it was *in consequence* of the badness of his Microscope.' If those who observe, with however humble an instrument, will but rigidly observe the rule of recording only what they can *clearly see*, they can neither go far astray themselves, nor seriously mislead others.

Among the erroneous tendencies which Microscopic inquiry seems especially fitted to correct, is that which leads to the estimation of things by their merely sensuous or material greatness, instead of by their value in extending our ideas and elevating our aspirations. For we cannot long scrutinize the 'world of small' to which we thus find access, without having the conviction forced upon us, that all *size* is but relative, and that *mass* has nothing to do with real grandeur. There is something in the extreme of minuteness, which is no less wonderful,—might it not almost be said, no less majestic?—than the extreme of vastness. If the mind loses itself in the contemplation of the immeasurable depths of space, and of the innumerable multitudes of stars and systems by which they are peopled, it is equally lost in wonder and admiration, when the eye is turned to those countless multitudes of living beings which a single drop of water may contain, and when the attention is given to the wondrous succession of phenomena which the life-history of every individual among them exhibits, and to the order and constancy which this presents. Still more is this the case, when we direct our scrutiny to the penetration of that universe which may be said to be included in the body of Man, or of any one of the higher forms of organized being, and survey the innumerable assemblage of elementary parts, each having its own independent life, yet each working in perfect harmony with the rest, for the completion of the wondrous aggregate which the life of the whole presents. In the study of the one class of phenomena, no less than in the survey of the other, we are led towards that Infinity, in comparison with which the greatest and the least among the objects of Man's regard are equally insignificant; and in that Infinity alone can we seek for a Wisdom to design, or a Power to execute, results so vast and so varied, by the orderly co-operation of the most simple means."

In the chapter on the Construction of the Microscope, the Simple Microscope, Gairdner's Simple Microscope, Field's, Quekett's Dissecting Microscope, Field's Compound, Highley's

Compound, Nachet's, Smith and Beck's Student and Dissecting, Warrington's Universal, Ross's large Compound, Powell and Lealand's, and Smith and Beck's large Compound Microscope are all elaborately described by the author. "Without any invidious comparison," he remarks, "it may be safely said that whoever desires to possess a *first-class* Microscope, cannot do better than select one of the three last mentioned instruments." Of the patterns of microscopes of simple construction, capable, however, of answering every essential purpose, that of Smith and Beck's Student Microscope appears to the author (to say the least,) among the best. It is his own working instrument.

The Appendix, which is by the American Editor, contains the Application of the Microscope to Clinical Medicine, &c., all mention of which is omitted by the author for reasons above detailed. "Free use," the Editor remarks, "has been made of the excellent manuals of Beale and Bennett; and the various kindred treatises and journals have also been drawn upon." A general view of the subjects most required by students, is all that is claimed in this portion of the work. A short account of American Microscopes is also added, among which are given Spencer's Trunnion, Queen's, J. & W. Grunow's Student, and the inverted Microscope of Dr. J. Lawrence Smith, for particular descriptions of which we refer our readers to the work itself. The Appendix is illustrated by upwards of 100 wood engravings. The Editor's additions add greatly, in our opinion, to the value of the work.

In conclusion, we reiterate our recommendation of Dr. Carpenter's work as a most interesting and instructive work, full of information, agreeably told; to all who take an interest in the subjects it treats of, it will be found of the greatest assistance.

The American Edition is printed on excellent paper and is beautifully illustrated. We are pleased to see, also, that it is reprinted with the author's sanction.

---

*Transactions of the State Medical Society of New York.* 1856.

The opening paper of the Transactions is a very able and eloquent "Eulogium upon the life and character of Theodric Romeyn Beck, M. D., LL. D.," by Frank H. Hamilton, M. D.,

President of the Society. Although Dr. Beck, at the early age of twenty-seven, formally relinquished the practice of medicine, having accepted the appointment of Principal to the Albany Academy, "yet his interest in the science did not cease, but to the improvement and perfection of some one or other of its departments, the balance of his life was, in a great measure, devoted," in proof which we may instance his celebrated work on Medical Jurisprudence. Did our space permit we would gladly dwell on many points in the life and writings of this eminent physician. He died on the 19th of November, 1855, at the age of sixty-four years and three months, the immediate cause of his death being a "defect in his power to assimilate."

The second article in the Transactions is a "Report on Tuberculosis and Tubercular Pneumonia," by C. B. Coventry, M. D. After a few remarks on the importance of the subject, Dr. Coventry asks, What is Tuberculosis? What Tubercular Phthisis? and answers the question by citing the opinions of Williams, Henry Ancell, Von Virchow, &c.; the "most satifactory observations on the character and development of tubercles" he deems to be those recently made by Dr. Radcliff Hall. The causes, symptoms and treatment of the affection are discussed in separate chapters; but we doubt, were we to transfer the whole to our pages, if we would communicate much to our readers that is not already familiar to the profession.

Dr. Blatchford's report on rest and the abolition of pain in the treatment of disease is an excellent paper, that will well repay a perusal; as will also Dr. March's article on Encysted Osseous Tumors. The diagnosis and treatment of these tumors are well described, and two cases are related which occurred in the writer's practice.

In Dr. Townsend's paper on "Malignant Pustule and Scrofulous Gangrene, is given the history of three cases of the former disease, occurring in three men who had been engaged a few days previously in slaughtering a diseased cow (intending to sell it), the flesh of which was, in many places, to use their own expression, "as black as their hats." One of the men died with tetanus, and the woman who washed his clothes "had afterwards a small pustule on her hand, similar in all respects to those upon the arms of the men."

We have carefully read the remaining papers, but failed to discover anything of special interest, if we except the essay on "Fetation from Coition to Parturition," by Dr. Thomas Goodsell, a gentleman on whose brow the snows of eighty winters have fallen. We recommend the perusal of the essay to those who are anxious, to use the language of the author, to "get a sly peep into nature's secret laboratory through an outer door inadvertently left a little ajar, when we apparently caught Dame Nature at her work."

The following which we extract from the President's Inaugural Address is not without interest. "Both licensed and unlicensed practitioners are thus (sec. 26) made liable in a civil action for damages in case of mal-practice ; but it is only the unlicensed practitioners (sec. 27) who, in the same case, are made liable in a criminal action for *misdemeanor*, and who, upon conviction, *may be imprisoned in a county jail.*" The law of New York having made this distinction between licensed and unlicensed practitioners; it being also "the intention of our legislators that no licenses obtained in other countries, or in other States of the Union, should constitute a license to practice in their State," it becomes a duty incumbent upon the county societies, many of which have suspended their annual meetings, to continue their organizations, as the law would otherwise bear very oppressively and unequally upon a portion at least of the profession. The Transactions, we observe, are published at the State's expense.

---

*History of the Ligature applied to the Brachio-Cephalic Artery, with Statistics of the Operation.* (Paper read before the Tennessee State Medical Society, May, 1856.) By PAUL F. EVE, M. D.

A very excellent paper, in which the history of all the cases on record, commencing with Dr. Mott's celebrated operation, is fully related. The following table exhibits the invariable fatal nature of this operation :

STATISTICS OF ATTEMPTS TO OBLITERATE THE BRACHIO-CEPHALIC.

| Surgeon. | Year. | Age. | Sex. | Result and Cause of Death. |
|---|---|---|---|---|
| 1. Mott, | 1818 | 57 | Male | Death on the 26th day from repeated hemorrhage. |
| 2. Graefe, | 1822 | adult | " | Death on 65th day from hemorrhage. |
| 3. Norman, | 1824 | " | | Death. |
| 4. Arendt, | 1826 | | | Death on the 8th day from inflammation of sac, pleura and lung. |
| 5. Hall, | 1830 | " | " | Death on the 6th day from dyspnœa and hemorrhage. |
| 6. Bland, | 1832 | 31 | " | Death on the 18th day from repeated hemorrhage. |
| 7. Bujalski, | before 1840 | | | Death on the 2d or 3d day. |
| 8. Bujalski, | " | | | Death on the 2d or 3d day. |
| 9. Lizars, | 1836 | 30 | " | Death on the 21st day from repeated hemorrhage. |
| 10. Dupuytren, | | | | |
| 11. Hutin, | 1842 | adult | " | Death the 11th hour; antecedent hemorrhage and exhaustion. |
| 12. Porter, | 1831 | | | No ligature, patient recovered. |
| 13. Kuhl, | 1836 | 43 | | Death on 3d day of hemorrhage. |
| 14. Liston, | 1838 | adult | Male | Death on the 13th day. |
| 15. Key, | 1844 | " | Female | Failed, yet patient died of pulmonary distress and exhaustion. |
| 16. Hoffman, | about 1840 | | | Died. |

"In reality the ligature was tied around the innominata in only ten of these cases, viz., Mott's, Graefe's, Norman's, Arendt's, Bland's, Bujalski's, Bujalski's, Lizars', Dupuytren's and Hutin's. In Hall's, the ligature was passed through the artery; in Kuhl's and Liston's the carotid and subclavian were tied just beyond the bifurcation; and in Porter's, Key's and Hoffman's the operation was abandoned and no ligature employed.

In every case where a ligature was applied either to the brachio-cephalic, or near its division into right subclavian and right carotid, *i. e.*, thirteen cases, death has followed; even in two where the operation was abandoned, there was a fatal result in one; and in the sixteen cases one alone recovered, and in that no ligature was used, the vessel having been simply exposed; the cure in this case was spontaneous, and in all probability entirely independent of the operation.

In this collection, embracing sixteen cases in which the ligature was attempted to the arteria innominata, I have given, as far as I could obtain it, the history of each one. Other similar operations may have been made, but these are all that are found on record.

In conclusion, after this exposition, let me ask, who will again venture *to tie the brachio-cephalic artery?*"

*Headaches, their Causes and their Cure.* By HENRY G. WRIGHT, M. D., M. R. C. S. L., &c. New York: S. S. & W. Wood. 1856.

This is a very excellent brochure, affording a good deal of information upon a subject which most works say but little about. Headaches in childhood and youth, in adult life and old age are severally described; the largest portion of the work, however, being very properly taken up with the description of the numerous varieties of the affection which occur during the middle periods of life. The headaches of this period are described under five heads, as being either dependant on the circulating system; on the digestive organs; on the nervous system; as rheumatic: or as headaches dependant on organic disease. The diagnosis, pathology, and treatment of all these forms are, on the whole, well described; though, we think, with more labor and research, a much more complete and satisfactory work might have been given us. Tables of formulæ are added at the end of the book.

---

*The Dissector's Manual of Practical and Surgical Anatomy.* By ERASMUS WILSON, F. R. S., &c. *The third American from the last revised London Edition. Illustrated with one hundred and fifty-four wood engravings.* Edited by WM. HUNT, M. D., Demonstrator of Anatomy in the University of Pennsylvania. Philadelphia: Blanchard & Lea. 1856.

We are glad to see another edition of this favorite Dissector, the best, in our opinion, in the English language. The present edition is considerably enlarged and much improved.

